# Artificial intelligence for translational personalized neoantigen cancer vaccine development

**DOI:** 10.1186/s12929-026-01286-3

**Published:** 2026-08-31

**Authors:** Chun-Yu Wei, Hsuan-Chao Lin, Chang-Jiun Wu, Chun-Nan Kuo, Che-Mai Chang, Wan-Hsuan Chou, Sheng-Po Chou, Sheng-Hsiang Feng, Wan-Chen Huang, Shisong Jiang, Benjamin P. Fairfax, Kang-Yun Lee, Wei-Chiao Chang

**Affiliations:** 1 Core Laboratory of Neoantigen Analysis for Personalized Cancer Vaccine, Office of R&D, Taipei Medical University, Taipei, 110 Taiwan; 2 Department of Clinical Pharmacy, School of Pharmacy, Taipei Medical University, Taipei, 110 Taiwan; 3 Department of Pharmaceutical Sciences, School of Pharmacy, Taipei Medical University, Taipei, 110 Taiwan; 4 Department of Pharmacy, Wan Fang Hospital, Taipei Medical University, Taipei, 116 Taiwan; 5 Master Program in Clinical Genomics and Proteomics, School of Pharmacy, Taipei Medical University, Taipei, 110 Taiwan; 6 Department of Pharmacy, Taipei Medical University Hospital, Taipei Medical University, Taipei, 110 Taiwan; 7 Taipei Cancer Center, Taipei Medical University Hospital, Taipei Medical University, Taipei, 110 Taiwan; 8 Single-Molecule Biology Core Lab, Institute of Cellular and Organismic Biology, Academia Sinica, Taipei, Taiwan; 9 Department of Oncology, University of Oxford, Oxford, UK; 10 MRC Weatherall Institute of Molecular Medicine, University of Oxford, Oxford, UK; 11 Division of Pulmonary Medicine, Department of Internal Medicine, Wan Fang Hospital, Taipei Medical University, Taipei City, 116 Taiwan; 12 Division of Pulmonary Medicine, Department of Internal Medicine, School of Medicine, College of Medicine, Taipei Medical University, Taipei, 110 Taiwan; 13 TMU Research Center of Thoracic Medicine, Taipei Medical University, Taipei, 110 Taiwan; 14 School of Pharmacy, College of Pharmacy, Kaohsiung Medical University, Kaohsiung, 807 Taiwan

**Keywords:** Neoantigen cancer vaccine, Artificial intelligence, Personalized immunotherapy, Human leukocyte antigen, T-cell receptor, Lipid nanoparticle, Translational oncology

## Abstract

Personalized neoantigen cancer vaccine is a promising strategy for precision immunotherapy by targeting patient-specific and mutation-derived tumor antigens. Early clinical studies have demonstrated the feasibility, safety, and immunogenicity of these vaccines across multiple solid tumors, with encouraging outcomes particularly when combined with immune checkpoint blockade. However, broader clinical translation remains limited by sequential bottlenecks across the vaccine development pipeline, including false-positive neoantigen selection,  imperfect modeling of antigen processing and HLA presentation, limited prediction of T-cell receptor recognition, and challenges in formulation, delivery, and manufacturing. Artificial intelligence and advanced computational workflows are increasingly integrated into this pipeline to improve candidate prioritization and support more reproducible decision-making. In this review, we summarize clinical progress and key translational barriers in personalized neoantigen vaccination, and discuss how AI-enabled approaches may contribute across four major stages: multi-omics integration for neoantigen discovery, processing-aware HLA presentation prediction, structure-aware and TCR-informed immunogenicity modeling, and data-driven formulation optimization, particularly for lipid nanoparticle-based delivery systems. These approaches are able to help narrow biological and chemical search spaces, improve prioritization, and provide mechanistic insights into antigen presentation and immune recognition rather than replacing experimental validation. This articlefurther addresses future implementation challenges, including dataset diversity, model interpretability, prospective benchmarking, manufacturing traceability, and evolving regulatory frameworks for individualized mRNA cancer immunotherapies. Integrating computational innovation with rigorous immunological validation, scalable manufacturing, and regulatory oversight will be essential for advancing personalized neoantigen vaccines toward broader clinical implementation.

## Background

Cancer immunotherapy has transformed the treatment landscape of multiple malignancies, particularly through the clinical use of immune checkpoint blockade. Nevertheless, only a subset of patients achieves durable responses, and many tumors remain poorly responsive because of the limited pre-existing antitumor immunity, low immunogenicity, immune escape, or an immunosuppressive tumor microenvironment. These limitations have driven interest in therapeutic cancer vaccines capable of actively inducing tumor-specific immune responses [1, 2].

Among emerging vaccine strategies, personalized neoantigen cancer vaccines represent a particularly attractive approach. Neoantigens arise from tumor-specific somatic alterations which provide a strong biological rationale for selective immune targeting while potentially reducing the risk of central tolerance and off-target autoimmunity. Advances in next-generation sequencing, human leukocyte antigen (HLA) typing, immunopeptidomics, and synthetic vaccine platforms have made it increasingly feasible to identify patient-specific candidate neoantigens and incorporate them into individualized vaccine products [1–5]. Despite this promise, the clinical translation of personalized neoantigen vaccines remains constrained by substantial biological and logistical attrition. Only a small subset of tumor mutations ultimately give rise to naturally presented and immunogenic T-cell targets indicating that current prediction pipelines still incompletely capture the biological steps linking mutation, antigen processing, HLA presentation, and T-cell recognition [3–6]. At the same time, manufacturing patient-specific vaccines, particularly mRNA- and lipid nanoparticle-based formulations, requires rapid, robust, and reproducible integration of multi-omics and formulation-related information within clinically actionable timelines [7].

To address these challenges, artificial intelligence (AI) and machine learning approaches are increasingly being explored as decision-support systems in neoantigen vaccine development. Rather than replacing experimental validation, advanced computational workflows may help integrate heterogeneous biological datasets, improve candidate prioritization, support the modeling of antigen presentation and immune-recognition-related features, and guide data-driven formulation optimization. AI may help narrow biological and chemical search spaces and enable more reproducible prioritization across the vaccine development pipeline [8, 9]. In this review, we summarize recent clinical progress in personalized neoantigen cancer vaccines and examine the sequential bottlenecks that continue to limit their broader implementation. We then discuss how AI-enabled methodologies contribute across key intervention points, from multi-omics neoantigen discovery and antigen presentation prediction to T-cell recognition modeling and formulation design. Finally, we address the implementation challenges, including dataset diversity, model explainability, manufacturing traceability, and evolving regulatory guidance for individualized cancer immunotherapies.

### Clinical progress and translational promise of neoantigen cancer vaccines

#### Early clinical evidence across tumor types

**Fig. 1:**
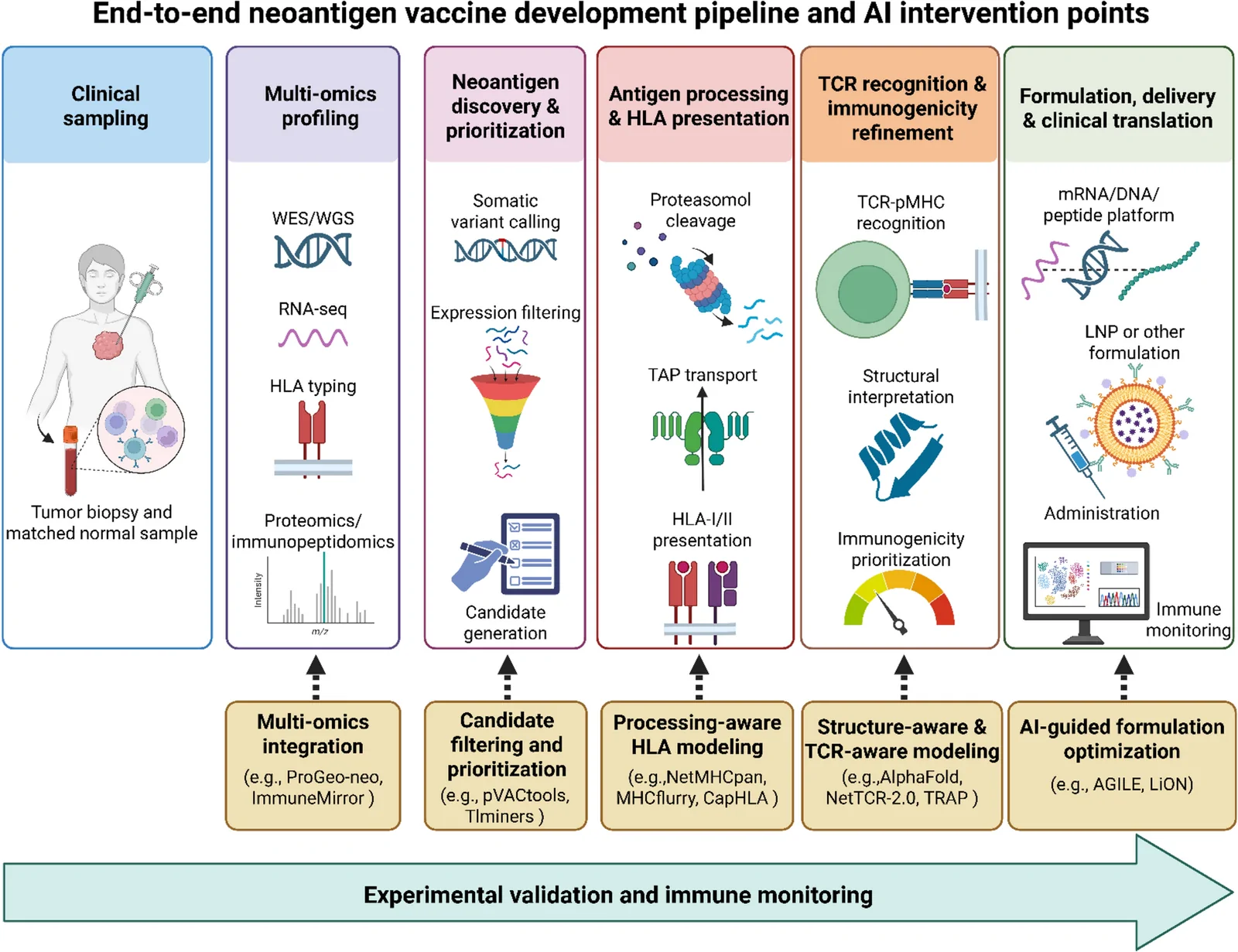
End-to-end neoantigen vaccine development pipeline and AI intervention points. Theneoantigen vaccine development workflow begins with clinical sampling and multi-omics profiling, followed by neoantigen discovery and prioritization, antigen processing and HLA presentation prediction, T-cell receptor (TCR) recognition refinement, and vaccine formulation and clinical translation. Across this pipeline, AI-enabled and computational approaches can support multi-omics integration, candidate filtering and prioritization, processing-aware HLA modeling, structure-aware immune recognition modeling, and formulation optimization. Representative tools are shown at selected steps, while experimental validation and immune monitoring remain essential throughout the workflow (This figure was generated by BioRender)

In recent years, neoantigen-based cancer vaccines have advanced rapidly through clinical development, with early-phase studies establishing feasibility and immunogenicity across multiple solid tumors and pivotal phase 3 trials now underway in selected disease settings. The end-to-end development of personalized neoantigen vaccines involves sequential steps from clinical sampling and multi-omics profiling to antigen prioritization, vaccine formulation, and clinical administration (Fig. 1). For example, the INTerpath-001 phase 3 clinical trial in resected high-risk melanoma has an estimated enrollment of approximately 1089 patients, underscoring the growing clinical maturity of this therapeutic strategy (NCT05933577). Neoantigens are tumor-specific and therefore offer the conceptual advantage of minimizing central immune tolerance while reducing the risk of off-target autoimmunity [10, 11]. Early clinical studies have evaluated a broad range of personalized vaccine platforms, including lipid nanoparticle (LNP)-encapsulated mRNA vaccines, RNA mutanome approaches, peptide-based strategies, DNA-based constructs, viral vector-based approaches, and dendritic cell-based vaccines [12–18]. Trials have been conducted not only in immunologically active tumors such as melanoma, but also in more challenging disease settings, including pancreatic ductal adenocarcinoma and hepatocellular carcinoma [13, 16, 19]. Taken together, these studies indicate that personalized neoantigen vaccination is clinically feasible across diverse tumor contexts and can be integrated into contemporary immuno-oncology treatment paradigms [18–20]. Representative clinical studies supporting these observations are summarized in Table 1 .

**Table 1:** Clinical and translational evidence from personalized neoantigen vaccine studies

Vaccine/tumor setting	Phase/setting	Format	Key signal	Screened, n	ITT, n	Product release rate*	Turnaround time	Vaccinated, n/N (%)	Neoantigens selected	Epitope-level T-cell response	Patient-level response breadth	Ref
mRNA-4157/V940 (resected high-risk melanoma)	Phase IIb/adjuvant	Personalized mRNA-LNP	18-month RFS was 79% with V940 plus pembrolizumab vs 62% with pembrolizumab alone; HR 0.561	224	157 (107 combination; 50 pembrolizumab)	>99% (106/107)	~6–8 weeks	104/107 (97%)	Up to 34; 91% received 34; range 9–34	NA	NA	[16]
mRNA-4157/V940 (HPV-negative recurrent/metastatic HNSCC)	Phase I/CPI-naïve recurrent or metastatic disease	Personalized mRNA-LNP + pembrolizumab	ORR was 27.3% (6/22) and DCR was 63.6% (14/22) with INT plus pembrolizumab; sustained de novo T-cell induction was observed in 5/5 patients with available samples	NR; 28 enrolled/received pembrolizumab	28	NR as formal release rate; 6/28 discontinued before INT for clinical reasons	NR as formal manufacturing TAT; pembrolizumab 6-week lead-in before INT	22/28 (78.6%)	Up to 34	NR at epitope level	5/5 patients with available samples showed sustained de novo T-cell induction to targeted neoantigens	[84]
Autogene cevumeran/RO7198457 (resected PDAC)	Phase I/adjuvant after surgery	Personalized uridine mRNA-lipoplex vaccine + atezolizumab, followed by mFOLFIRINOX	Neoantigen-specific T-cell responses in 8/16 patients; responders showed longer RFS at 18 months and 3.2-year follow-up, with long-lived CD8⁺ T-cell clones	34 screened/consented; 32 enrolled	19 safety-evaluable; 16 vaccine/biomarker-evaluable	NR as formal release rate; 1/19 non-manufacture due to insufficient neoantigens	Median 9.4 weeks from surgery to vaccine administration (range 7.4–11.0)	16/19 atezolizumab-treated patients (84%); 16/34 screened (47%)	Up to 20	11% (25/230 neoantigens)	8/16 patients had neoantigen-specific T-cell responses; 4/8 responders had polytope responses	[19, 85]
EVX-01 (metastatic melanoma)	Phase I/metastatic disease, first dose-level cohort	Personalized synthetic peptides + CAF^®^09b, with anti-PD-1 CPI	EVX-01 was manufactured within 48–55 days and induced T-cell responses in all 5 evaluable patients; BOR included 1 CR and 2 PR	NR; 8 enrolled	5 treated/evaluable	100% (5/5 evaluable productions completed)	48–55 days manufacturing; first vaccination 51–60 days from baseline biopsy	5/8 enrolled (63%); 4/5 completed all 6 vaccinations	5–10	74% (23/31 neopeptides)	5/5 patients; 3–7 peptide-specific responses per patient	[22]
IVAC Mutanome/RNA mutanome vaccine (melanoma, stage III/IV)	Phase I/advanced or high-risk disease	Personalized poly-neo-epitope RNA, intranodal	All vaccinated patients developed poly-specific mutation-directed T-cell responses; two vaccine-related objective responses were reported in patients with metastatic disease	20	16 enrolled	100% (13/13)	103 days from mutation selection to vaccine release (range 89–160)	13/16 enrolled (81%)	Up to 10 mutations per patient; P09 received 5	60% (75/125)	100% (13/13; ≥3 mutations per patient)	[12]
GNOS-PV02 (advanced HCC)	Phase I/II/second-line advanced HCC	Personalized DNA plasmid vaccine + pIL12 + electroporation; with pembrolizumab	ORR 30.6% by RECIST 1.1; 3/36 CR; neoantigen-specific T-cell responses in 19/22 evaluable patients	52 assessed	36 mITT	100% (36/36 available for dosing)	NR; manufactured during first-line therapy and available by second-line eligibility	36/36 (100%)	Up to 40 neoantigens	Median 64.0% of encoded epitopes after treatment (range 0–100%)	19/22 (86.4%) immune-evaluable patients	[13]
Neo-DCVac (advanced lung cancer)	Pilot study/heavily pretreated advanced lung cancer	Personalized neoantigen peptide-pulsed autologous DC vaccine	ORR 25%; DCR 75%; PBMC responses to predicted neoantigen peptides in 10/10 immune-evaluable patients	18 recruited	12 enrolled/vaccinated	100% (12/12 feasible manufacturing)	Median 2.8 months from biopsy to first vaccination (range 2.1–3.5)	12/18 recruited (67%); 12/12 enrolled (100%)	12–30 peptide-based neoantigens per patient	NR as cohort-level proportion; ≥8 responsive neoantigens in 6/10 immune-evaluable patients	10/10 immune-evaluable patients	[17]
NEO-PV-01 (advanced non-squamous NSCLC)	Phase Ib/first-line advanced or metastatic disease	Personalized synthetic long peptides + poly-ICLC, with pembrolizumab + chemotherapy	ORR 34% in ITT and 69% in vaccinated patients; neoantigen-specific CD4⁺/CD8⁺ responses and epitope spreading, including mutant KRAS responses, were observed	NR; 38 enrolled/ITT	38	NR as formal release rate; 10/38 not vaccinated due to inadequate tumor and/or insufficient neoantigens	NR; vaccination initiated at week 12 after 4 cycles during vaccine production	21/38 (55%); 16/21 completed vaccination	Up to 20	Average 55% of vaccine epitopes; 204 peptides tested across 13 patients	13/13 immune-evaluable patients; epitope spread in 9/13	[21]
NEO-PV-01 multi-tumor phase Ib trial (melanoma, NSCLC, and bladder cancer)	Phase Ib / metastatic or unresectable disease	Personalized synthetic long peptides + poly-ICLC, with nivolumab	Across vaccinated melanoma, NSCLC, and bladder cancer cohorts, ORRs were 59%, 39%, and 27%, respectively; de novo neoantigen-specific CD4⁺/CD8⁺ T-cell responses were detected in all immune-evaluable patients	NR; 82 enrolled/ ITT	82	NR as formal release rate	NR as formal manufacturing TAT; vaccination began at week 12 while vaccines were generated	60/82 (73%)	Up to 20	Average 42% CD4⁺ and 24% CD8⁺ responses among vaccinating peptides; 570 peptides tested across 34 immune-evaluable patients	34/34 immune-evaluable patients; epitope spread in 16/25 evaluable patients	[14]
NeoVax (resected high-risk melanoma)	Phase I/ adjuvant after resection; long-term follow-up	Personalized synthetic long peptides + poly-ICLC	Initial study showed polyfunctional CD4⁺/CD8⁺ responses to 58/97 and 15/97 vaccine neoantigens; long-term follow-up showed persistent memory T-cell responses and epitope spreading	NR; 12 enrolled	12 enrolled	NR as formal release rate; GMP peptides prepared for 8 vaccinated patients	Median 18 weeks from surgery to vaccine administration in initial cohort	8/12 enrolled (67%)	Up to 20	Initial: CD4⁺ 60% (58/97 IMP), CD8⁺ 16% (15/97 IMP); follow-up: CD4⁺ 56% (69/124 IMP), CD8⁺ 13% (15/117 IMP)	Initial: 6/6 vaccinated; follow-up: persistent responses in 8/8 vaccinated patients	[18, 27]
NeoVax (MGMT-unmethylated glioblastoma)	Phase I/Ib /adjuvant after surgery and radiotherapy	Personalized synthetic long peptides + poly-ICLC	Polyfunctional neoantigen-specific CD4⁺/CD8⁺ T-cell responses and increased intratumoral T cells were observed in patients not receiving dexamethasone	NR; 10 enrolled	10	8/10 (80%) advanced to vaccination	Median 19.9 weeks from surgery to first vaccination (range 17.1–24.7)	8/10 (80%)	Median 12 peptides (range 7–20)	NR as a proportion; multiple responses detected in patients 7–8	2/5 immune-evaluable boosted patients; 2/2 without dexamethasone	[86]
NeoVax / PCV (high-risk resected clear cell RCC)	Phase I/adjuvant after resection	Personalized synthetic long peptides + poly-ICLC ± local ipilimumab	No RCC recurrence in 9/9 patients at median 40.2-month follow-up; T-cell responses in all patients; autologous tumour reactivity in 7/9	10 assessed	9 enrolled/analysed	100% (9/9 successfully manufactured)	NR	9/9 enrolled (100%; 9/10 assessed)	Median 15 peptides per patient (range 8–19); up to 20 planned	47% (61/129 peptides); driver 65% (11/17), passenger 44% (50/112)	9/9 patients; median 7 reactive peptides per patient (range 1–14)	[20]
NOUS-PEV (metastatic melanoma)	Phase Ib / first-line metastatic melanoma	Personalized viral-vector prime-boost, GAd20/MVA + pembrolizumab	Neoantigen-specific T-cell responses in all immune-evaluable full prime-boost patients; BOR among vaccinated patients: 1 CR, 3 PR, 1 SD, 1 PD	NR; 7 enrolled	7 enrolled	11/12 vaccines released/administered; reported as ~90%	~8 weeks from biopsy; GAd20 mean 59 days, MVA mean 81 days	6/7 enrolled (86%)	Up to 60 neoantigens; Pt6 encoded 34	Pool-level: 71% (15/21 pools); individual epitope NR	4/4 immune-evaluable full prime-boost patients	[15]

*BOR* best overall response, *CPI* checkpoint inhibitor, *CR* complete response, *DCR* disease control rate, *DC* dendritic cell, *HCC* hepatocellular carcinoma, *HNSCC* head and neck squamous cell carcinoma, *IMP* immunizing peptide, *INT* individualized neoantigen therapy, *ITT* intention-to-treat, *LNP* lipid nanoparticle, *mITT* modified intention-to-treat, *NA* not available or not applicable, *NR* not reported, *NSCLC* non-small cell lung cancer, *ORR* objective response rate, *PBMC* peripheral blood mononuclear cells, *PDAC* pancreatic ductal adenocarcinoma, *PFS* progression-free survival, *PR* partial response, *RCC* renal cell carcinoma, *RFS* recurrence-free survival, *SD* stable disease, *TAT* turnaround time

^*^Product release rate refers to released or successfully manufactured vaccine products among initiated or evaluable manufacturing attempts, when reported. Clinical attrition before vaccination was not counted as product release failure unless the study explicitly reported a manufacturing or product-release failure. Denominators for vaccination, epitope-level T-cell response, and patient-level response breadth vary across studies; direct cross-trial comparison should therefore be interpreted cautiously. Epitope spreading, when included, refers to post-vaccination responses against non-vaccine neoantigens and is distinct from direct vaccine-specific response breadth

### What has been established so far

Several important proof-of-concept milestones have emerged from these early clinical experiences. First, the logistical feasibility of individualized vaccine development has been demonstrated in prospective clinical studies, including tumor sequencing, neoantigen selection, and the manufacturing of patient-specific products containing multiple neoepitopes [16, 19, 21]. Manufacturing timelines vary across platforms, typically requiring 7–8 weeks for peptide-based EVX-01 [22] and about 8 weeks for the viral vector-based NOUS-PEV platform [15]. Second, the overall safety profile has been favorable, with most treatment-related adverse events being mild to moderate, such as injection-site reactions, fatigue, and chills, whereas severe vaccine-related toxicities have been reported only infrequently [13, 16, 19]. In the KEYNOTE-942 trial, the incidence of grade ≥3 treatment-related adverse events was higher in the combination arm (25%) than in the pembrolizumab monotherapy arm (18%). Importantly, no grade 4–5 adverse events were attributed to the mRNA-4157 vaccine [16]. Third, these studies have consistently shown that neoantigen vaccines can induce detectable neoantigen-specific T-cell responses, supporting their immunogenicity across different platforms and disease settings [12, 18, 19]. For example, NEO-PV-01 studies reported post-vaccination neoantigen-specific CD4^+^ and CD8^+^ T-cell responses across melanoma, NSCLC, and bladder cancer cohorts, with subsequent NSCLC-focused data further supporting the feasibility and immunogenicity of this platform [14, 21]. Early signals of clinical activity have also been reported, particularly in combination with immune checkpoint blockade. In KEYNOTE-942, V940 plus pembrolizumab was associated with an 18-month recurrence-free survival of 79% versus 62% with pembrolizumab alone (hazard ratio, 0.561) [16], while in pancreatic ductal adenocarcinoma, responders to autogene cevumeran showed markedly prolonged recurrence-free survival compared with non-responders (median not reached versus 13.4 months) [19]. Although these findings should still be interpreted as early-stage evidence rather than definitive confirmation of efficacy, they collectively establish neoantigen vaccination as a credible and translationally relevant therapeutic strategy [23].

### Key bottlenecks in neoantigen vaccine development

Despite encouraging clinical progress, several fundamental challenges continue to limit the efficacy and scalability of neoantigen-based cancer vaccines [19, 24]. These bottlenecks arise sequentially across the development pipeline, creating a compounding attrition effect in which limitations at one stage propagate and compromise downstream outcomes.

### Limited neoantigen quality and high false-positive rates

A major limitation remains the substantial discrepancy between the large number of genomic mutations identified and the small fraction that are truly immunogenic [19, 24]. Current selection pipelines frequently yield high false-positive rates, as sequence-based mutation identification does not inherently guarantee effective epitope production [24, 25]. This attrition is evident in recent clinical studies: in the autogene cevumeran pancreatic cancer trial, only 11% (25 of 230) of administered vaccine neoantigens elicited measurable T-cell responses [19, 26]. In the NeoVax melanoma study, while approximately 60% of vaccine neoantigens induced CD4⁺ T-cell responses, about 16% triggered CD8⁺ cytotoxic T-cell responses [27]. These findings illustrate a persistent gap between computationally selected candidates and neoantigens capable of inducing cytotoxic T-cell immunity. As a result, vaccine designs mainly based on conventional prioritization criteria may still include many peptides with limited functional relevance, potentially reducing the effective immunogenic content of the final vaccine product [19, 28].

### Imperfect modeling of antigen processing and presentation

Antigen presentation is a complex, multi-step biological process involving proteasomal cleavage, TAP-mediated transport, and peptide-HLA loading [6]. However, current computational frameworks often simplify this process into peptide-HLA binding prediction, without fully capturing upstream processing dynamics [3, 24]. Limitations in the sensitivity and coverage of immunopeptidomics further restrict the availability of high-quality training data, particularly across diverse tumor types and HLA backgrounds [29]. Consequently, many predicted candidates may never be naturally presented on the tumor cell surface. This is a critical attrition step recently quantified by findings that only approximately 22% of predicted neoepitopes are successfully presented on HLA molecules, thereby limiting their relevance as vaccine targets [30].

### Challenges in predicting T-cell recognition

Even when candidate epitopes are successfully processed and presented by HLA molecules, the capacity to T-cell responses remains difficult to predict. Peptide-HLA binding provides a prerequisite for presentation, but functional immunogenicity ultimately depends on productive engagement between the peptide-HLA complex and a cognate T-cell receptor (TCR) [19, 31]. The TCR-peptide-HLA interaction is governed by complex three-dimensional docking geometry, molecular flexibility, peptide conformation, and other biophysical properties that are incompletely captured by current predictive sequence- or affinity-based models alone [32–34]. Furthermore, the scarcity of paired TCR-pMHC datasets and the extreme diversity of the TCR repertoire limit model generalizability [35–37]. These limitations make it difficult to identify neoantigens that reliably induce CD8⁺ cytotoxic T-cell responses, which remains a major challenge in neoantigen vaccine development [24, 38].

### Translational and population-level constraints

Beyond biological constraints, neoantigen vaccine development also faces translational and population-level challenges. Individualized manufacturing often requires several weeks, creating a delay between tumor sampling and vaccine administration. During this interval, tumor evolution or changes in the immune microenvironment may reduce the relevance of the originally selected vaccine targets [39, 40]. In parallel, prediction models may be affected by inter-individual HLA diversity and the underrepresentation of non-European populations in training datasets. Whether these training imbalances consistently reduce accuracy for out-of-distribution HLA alleles remains under debate. Nevertheless, these imbalances raise concerns about model robustness and equitable performance, particularly for HLA alleles or haplotypes that are common in underrepresented populations but have limited representation in available training or validation datasets [41, 42]. In addition, delivery systems, including lipid nanoparticles and peptide-based formulations, remain subject to constraints related to stability, delivery efficiency, and scalability, contributing to variability in clinical outcomes [7, 40].

**Fig. 2:**
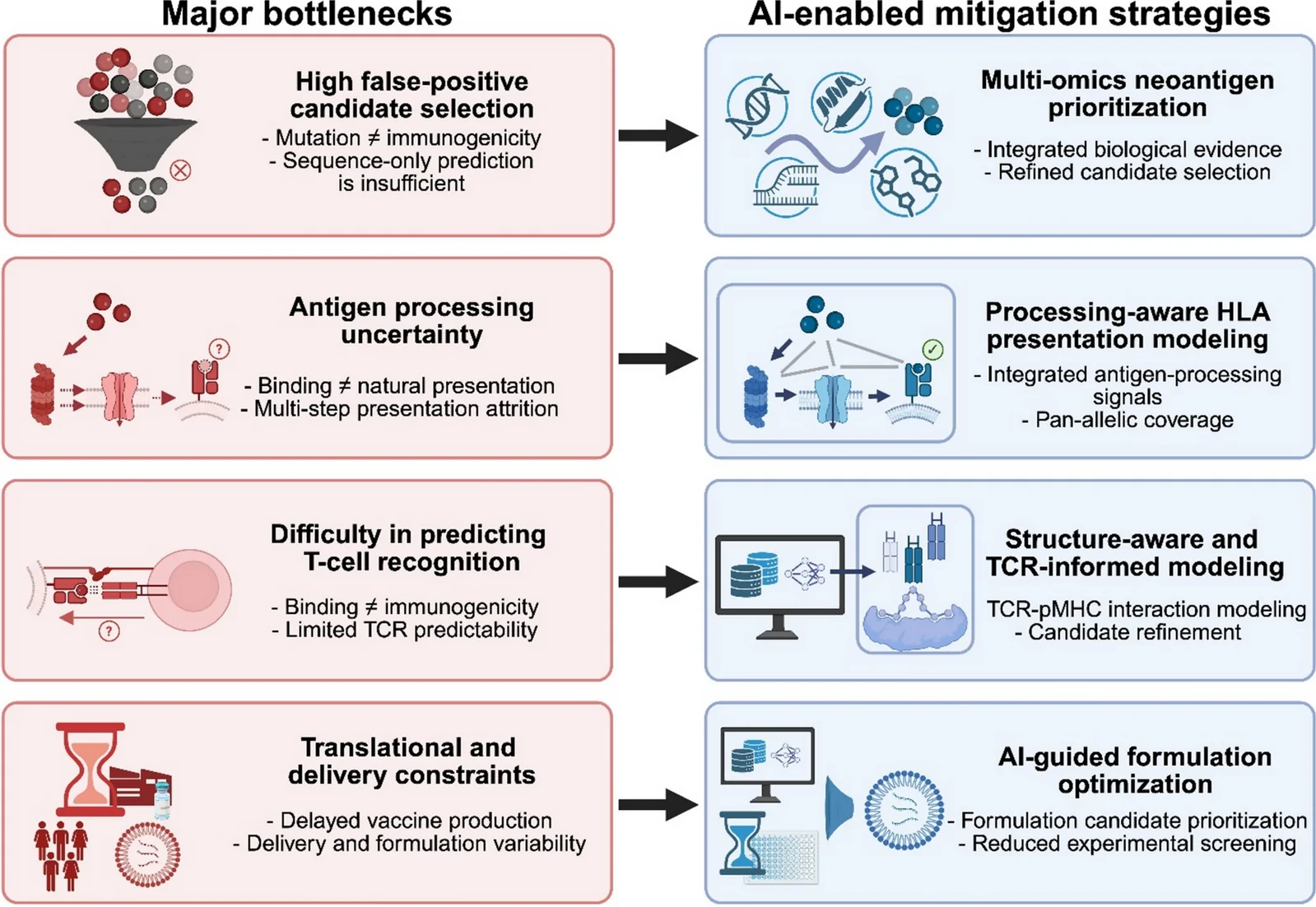
Major bottlenecks in neoantigen vaccine development and AI-enabled mitigation strategies. Neoantigen vaccine development is limited by false-positive candidate selection, incomplete antigen processing and presentation modeling, uncertain T-cell recognition, and translational constraints in manufacturing and delivery. AI-enabled approaches may help mitigate these bottlenecks through multi-omics prioritization, processing-aware HLA modeling, structure-aware TCR-informed prediction, and formulation optimization, while experimental and clinical validation remain essential (This figure was generated by BioRender

These challenges are closely linked across the neoantigen vaccine pipeline. Uncertainty introduced at one stage can carry forward and affect downstream decisions, from candidate prioritization to formulation and clinical development [43, 44]. This interdependence supports the use of integrated computational approaches, including AI-based methods, to improve coordination across the pipeline (Fig. 2).

### Artificial Intelligence across the neoantigen vaccine development pipeline

To address the sequential bottlenecks outlined above, machine learning and deep learning approaches are increasingly being incorporated into the neoantigen vaccine development pipeline [3, 38]. Rather than replacing experimental validation, these methods aim to reduce the search space, improve candidate prioritization, and support more reproducible integration of genomic, transcriptomic, proteomic, structural, and formulation-related data. In the following sections, we discuss how AI-enabled tools contribute to four major stages of the pipeline: multi-omics-based neoantigen discovery, antigen processing and HLA presentation prediction, TCR-aware immune recognition modeling, and vaccine formulation and delivery optimization (Fig. 1 and Table 2).

**Table 2:** Representative AI-enabled tools across the neoantigen vaccine development pipeline

Category	Tool/Model	AI/computational approach	Main input	Main output	Pipeline application	Key note/limitation	Ref
Multi-omics & Identification	ProGeo-neo	Proteogenomic integration workflow	Genomic, transcriptomic, and MS-based proteomic data	Candidate neoantigens with protein-level support	Candidate identification and biological filtering	Increases biological plausibility, but performance depends on proteomic depth and peptide detectability	[45, 46]
	ImmuneMirror	Machine learning-based integrative pipeline (balanced random forest)	Tumor mutation and expression-related data derived from WES/RNA-seq	Predicted and prioritized neoantigen candidates	Expression-aware candidate prioritization	Integrates sequencing-derived features with ML-based ranking, but adoption and external benchmarking remain more limited than widely used modular pipelines	[47]
	pVACtools	Modular computational pipeline	Somatic variants, HLA typing, and expression data	Ranked neoantigen candidates	End-to-end candidate generation and filtering	Widely used and practical, but final ranking remains dependent on underlying prediction modules	[49]
	TIminer	Integrated immunogenomics workflow	DNA/RNA sequencing and immune-related features	Integrated immunogenomic features, including HLA typing, neoantigen prediction, and immune context summaries	Immunogenomic profiling for candidate selection	Useful for standardized profiling, but not specifically optimized for all neoantigen vaccine settings	[48]
Peptide-HLA Presentation & Immunogenicity	NetMHCpan-4.0	Artificial neural network integrating binding affinity and eluted ligand data	Peptide sequence and HLA class I allele information	Predicted peptide-HLA class I binding affinity and presentation likelihood	HLA-I peptide presentation prediction	Widely used pan-allelic HLA-I predictor, but binding or presentation scores do not directly establish immunogenicity	[53]
	NetMHCIIpan-4.3	Artificial neural network (ANN)	Peptide sequence and HLA class II alleles	Predicted peptide-HLA class II binding/presentation likelihood	Presentation prediction	Strong pan-allelic utility, but binding does not directly equate to immunogenicity	[56]
	MHCflurry	Neural network-based class I binding/presentation predictor	Peptide sequence and HLA class I alleles	Binding affinity and presentation-related scores	Class I peptide-HLA prediction	Widely used and validated, but performance still reflects training-data coverage	[54]
	CapHLA	Convolution + attention	Peptide and HLA sequence information	Peptide presentation probability and binding affinity prediction	Pan-HLA presentation modeling	Designed to jointly model HLA-I and HLA-II presentation/binding, but broader independent benchmarking in vaccine-specific settings remains limited	[55]
	DeepImmuno	Convolutional neural network	Peptide-MHC-related features	Predicted immunogenicity score	Immunogenicity ranking	Extends beyond binding-only prediction, but training labels and negative-set definitions remain major constraints	[58]
	DeepNeo	Deep-learning models integrating peptide-MHC binding and T-cell reactivity modules	Peptide-HLA pairs and T-cell reactivity-related features	Predicted immunogenic neoantigen candidates	Candidate prioritization for vaccine design	Integrates MHC binding and T-cell reactivity prediction, but external clinical validation remains limited	[59]
	NeoDisc	Rule-based and machine-learning framework	Tumor genomic and immunologic data	Integrated neoantigen prioritization and antigen-presentation context assessment	Translational candidate selection	Best viewed as a prioritization framework rather than a single standalone predictor	[9]
	MUNIS	Bimodal deep learning framework (protein language model + LSTM)	HLA-I allele sequence, peptide sequence, and optional flanking residues	Predicted HLA-I peptide presentation score/epitope probability	Presentation-aware epitope prioritization	Jointly models HLA-I-peptide binding and antigen processing; strongest evidence is for HLA-I presentation prediction rather than direct TCR recognition	[57]
Structure-Aware & TCR Modeling	AlphaFold 3	Diffusion-based biomolecular complex structure prediction	Protein/complex sequence information	Predicted structures of biomolecular complexes	Structure-aware interpretation of pMHC/TCR interactions	Mechanistically informative, but not a stand-alone clinical prioritization engine	[63]
	AlphaFold-based modeling of NRAS neoantigen recognition	Structure-aware TCR-pMHC modeling	TCR, peptide, and HLA structural/sequence information	Modeled TCR-pMHC interaction features	Mechanistic interpretation of neoantigen recognition	Useful for structural interpretation, but does not replace functional validation	[62]
	NetTCR-2.0	Shallow convolutional neural network	Paired TCRα/β CDR3 sequence data and peptide information	Predicted TCR-peptide binding specificity	TCR-aware immune-recognition prediction	Requires sufficient high-quality paired TCRα/β data; generalization remains data-dependent	[64]
	DeepTCR	Deep representation learning	CDR3 sequences and V/D/J gene usage	TCR repertoire representations and specificity-related predictions	TCR repertoire modeling and immune-recognition analysis	Useful for repertoire-level modeling, but not a definitive neoantigen immunogenicity predictor	[65]
	TRAP	Contrastive learning-enhanced sequence/structure model	TCR, peptide, and MHC information	Predicted TCR-pMHC binding specificity	TCR-aware prioritization	More biologically expressive than binding-only models, but ground-truth datasets remain sparse	[66]
	SageTCR	Bi-level graph neural network (GNN)-based deep learning	TCR-pMHC structure	Predicted TCR-pMHC binding probability	TCR-aware prioritization	Strong dependence on high-quality input structures	[67]
Formulation & LNP Delivery	AGILE	Deep learning-guided lipid screening with combinatorial chemistry	Ionizable lipid libraries and delivery-performance data	Predicted high-performing ionizable lipid candidates	AI-guided ionizable lipid screening for mRNA delivery	Strong example of AI-assisted lipid discovery, but performance depends on experimental validation and context-specific delivery assays	[71]
	LiON	Directed message-passing neural network	Ionizable lipid structures, formulation/cargo/target metadata, and LNP activity datasets	Predicted lipid delivery performance and prioritized ionizable lipid candidates	AI-guided ionizable lipid discovery and LNP delivery optimization	Demonstrates deep learning-guided exploration of chemically diverse lipid design spaces; evidence is strongest in RNA delivery and pulmonary gene therapy settings rather than neoantigen cancer vaccine-specific applications	[73]

### Multi-omics integration for enhanced neoantigen discovery

To mitigate the high false-positive rates inherent in sequence-only predictions, AI-driven multi-omics frameworks integrate genomic, transcriptomic, and mass spectrometry (MS)-based proteomic data to enrich candidates with stronger evidence of expression and presentation. Machine learning models enable the integration of heterogeneous datasets, allowing prioritization of candidates supported by multiple layers of biological evidence. Representative approaches include ProGeo-neo, which leverages a proteogenomics strategy, directly combining MS proteomics with patient-specific genomic and transcriptomic profiles to confirm protein-level expression, thereby increasing the biological plausibility of identified candidates [45, 46]. Other frameworks, such as ImmuneMirror, utilize an integrative machine learning approach to combine whole-exome and RNA-sequencing data, prioritizing candidates that exhibit both high predicted MHC-binding affinity and substantial tumor tissue expression [47]. To streamline these complex data fusion processes, modular workflows like TIminer and pVACtools automate end-to-end immunogenomic profiling, ensuring reproducible candidate generation for clinical applications [48, 49].

Despite these advancements, multi-omics integration remains subject to challenges related to data quality and the dynamic nature of the tumor microenvironment. While these tools significantly narrow the pool of potential neoantigens, their performance remains constrained by sparsity and the limited sensitivity of MS-based detection for low-abundance peptides [29]. However, this persistent technical constraint highlights the need for continued refinement of both computational models and experimental validation strategies.

### Expanding beyond canonical mutation-derived neoantigens: noncanonical and dark-matter antigen sources

Personalized neoantigen vaccine pipelines have traditionally focused on canonical neoantigens arising from tumor-specific somatic single-nucleotide variants (SNVs) and small insertions or deletions. However, the antigenic landscape of cancer is broader than the conventional exonic mutanome. Tumor-specific or tumor-enriched antigens may also arise from noncanonical sources, including alternative open reading frames (altORFs), aberrant splicing, intron retention, fusion transcripts, circRNA-derived products, transposable elements, and endogenous retroviral elements [50]. These antigen classes are particularly relevant because they may not be captured by standard variant-calling pipelines and may instead require transcriptomic, proteogenomic, or immunopeptidomic evidence to support their expression, translation, and HLA presentation. Compared with many patient-private SNV-derived neoantigens, some noncanonical antigens, particularly those arising from recurrent splicing events or endogenous retroelements, may be shared across patients or tumor types, suggesting that these sources may inform both individualized and semi-shared vaccine strategies [51, 52].

AI-enabled and computational approaches may help incorporate these noncanonical sources into personalized vaccine workflows by addressing several linked tasks. First, transcriptome-aware models can support the reconstruction and prioritization of tumor-specific isoforms or aberrant splicing events, particularly when short-read RNA sequencing is complemented by long-read transcriptomic data. Second, models that integrate sequence context, coding potential, ribosome-profiling information, proteogenomic evidence, and normal-tissue expression atlases may help distinguish plausible tumor-specific translation products from transcriptional noise [9]. Third, immunopeptidomics-informed prioritization can help determine whether predicted noncanonical peptides are naturally presented by HLA molecules [52]. Because the discovery space for noncanonical antigens is large and may generate many candidate events, AI-based prioritization should be paired with tumor-specificity filtering and experimental validation before these candidates are considered functionally immunogenic.

### Advanced modeling of antigen processing and HLA presentation

Following multi-omics-based candidate filtering, a major next step is to improve the prediction of antigen processing and HLA presentation. Contemporary AI models increasingly move beyond peptide-HLA binding affinity alone by incorporating features related to proteasomal cleavage, TAP-mediated transport, and eluted ligand evidence. For HLA class I prediction, pan-allelic tools such as NetMHCpan are widely used to estimate peptide-HLA binding and presentation likelihood [53], whereas MHCflurry integrates antigen-processing signals into neural network-based models to improve class I presentation prediction [54]. To expand pan-allelic coverage across additional presentation contexts, CapHLA employs a convolution-attention mechanism to jointly model peptide presentation across both HLA class I and class II molecules [55], while NetMHCIIpan-4.3 leverages neural network models to address the open-binding-groove complexity of class II prediction [56]. Furthermore, bimodal frameworks are advancing the field by moving beyond linear sequences. MUNIS, for instance, integrates sequence-based representations derived from protein language models (PLMs) with advanced deep learning architectures, which has been reported to improve the prioritization of HLA class I-presented epitopes [57]. Integrative pipelines like NeoDisc combine rule-based and machine-learning approaches to assess the broader genomic context of antigen presentation [9]. To extend prediction beyond antigen presentation, tools such as DeepImmuno and DeepNeo use experimentally annotated datasets to prioritize candidates with greater immunogenicity or T-cell reactivity potential [58, 59].

Despite these robust modeling capabilities, current presentation predictors are inherently limited by the biases present in their training datasets. Because these models rely heavily on MS-derived immunopeptidomes, they frequently underperform when applied to rare HLA alleles or underrepresented tumor histologies. This data-dependency highlights a critical limitation that predictive accuracy often degrades for populations with diverse ethnic backgrounds, underscoring the pressing need for more globally representative training corpora [41, 42].

### AI opportunities for HLA class II and CD4⁺ T-cell neoantigen prediction

Although many neoantigen prediction pipelines have historically prioritized HLA class I-restricted epitopes and CD8⁺ cytotoxic T-cell responses, clinical studies of personalized neoantigen vaccines have repeatedly detected vaccine-induced CD4⁺ T-cell responses, in some cases more frequently than CD8⁺ responses. This observation suggests that CD4⁺ T cells should not be viewed merely as secondary bystanders in vaccine design. Through cytokine production, support of antigen-presenting cells, enhancement of CD8⁺ T-cell priming and memory formation, and remodeling of the tumor microenvironment, CD4⁺ T cells may contribute substantially to the breadth and durability of vaccine-induced antitumor immunity [60].

However, HLA class II neoantigen prediction presents distinct computational challenges. Class II molecules have open-ended binding grooves and accommodate peptides of variable length, often requiring identification of a shorter binding core within longer peptide sequences. In addition, HLA-DR, HLA-DQ, and HLA-DP molecules differ in the amount and quality of available training data, and peptide-HLA class II interactions are more promiscuous than typical class I binding events. These features make class II prediction more sensitive to dataset size, assay heterogeneity, negative-sample definition, and allele coverage. AI-enabled models may help by learning pan-allelic binding patterns, incorporating flanking sequence information, integrating eluted-ligand or immunopeptidomic evidence, and combining class II presentation predictions with tumor expression and CD4⁺ T-cell response data [61]. The performance of class II predictors should therefore be interpreted according to the endpoint being evaluated, ranging from binding affinity and natural presentation to functional CD4⁺ T-cell recognition. Nevertheless, the clinical value of AI-prioritized HLA class II neoantigens remains less established than HLA binding prediction itself, and prospective benchmarking against functional CD4⁺ T-cell responses will be essential.

### AI-driven modeling of TCR binding prediction

To address the challenges in predicting effective T-cell recognition, recent advances in AI-driven approaches have begun to move beyond peptide-HLA binding affinity by integrating structural and sequence-level features that more directly reflect T-cell recognition. While accurate modeling of antigen presentation represents a critical step, the ability of a presented epitope to elicit a functional cytotoxic T-cell response ultimately depends on its recognition by the highly diverse TCR repertoire. This additional layer of biological complexity contributes to the substantial attrition observed between predicted binders and truly immunogenic neoantigens, as reflected by the limited proportion of predicted neoantigens that ultimately elicit CD8⁺ T-cell responses [31, 32]. In this context, structure-aware modeling approaches have emerged as a complementary strategy to sequence-based prediction. Advances in deep learning-based protein structure prediction, particularly those enabled by models such as AlphaFold, have provided new opportunities to infer the three-dimensional conformations of peptide-HLA (pMHC) complexes and TCR-pMHC interfaces. These structural representations may offer insights into features such as peptide conformation, solvent exposure, and putative TCR contact residues, which are not readily captured by sequence-based models [32, 62, 63]. However, pMHC and TCR-pMHC modeling remains technically challenging, as peptide placement, MHC-specific conformational constraints, and flexible complementarity-determining regions can limit structural accuracy [8]. Therefore, structure-aware approaches are best viewed as tools for mechanistic interpretation and candidate refinement rather than stand-alone predictors of immunogenicity.

In parallel, machine learning models have been developed to estimate TCR-epitope or TCR-pMHC recognition. NetTCR-2.0 leverages shallow convolutional neural networks trained on paired TCRα/β CDR3 sequence data to predict TCR-peptide binding specificity [64], whereas DeepTCR applies deep representation learning to model TCR repertoires by jointly encoding CDR3 sequences and V/D/J gene usage [65]. A previous study demonstrated that a specialized pipeline utilizing the deep neural network AlphaFold can generate accurate structural models of T cell receptor-peptide-MHC complexes, thereby enabling the successful discrimination of true target peptide epitopes from decoy sequences with substantial accuracy [33]. More recent architectures, such as TRAP and SageTCR, have significantly advanced TCR-pMHC binding prediction by integrating sophisticated 3D structural features to capture subtle conformational dynamics and achieve superior predictive accuracy and generalizability in unseen-epitope settings over traditional sequence-only models [66, 67]. These approaches represent an important step toward modeling functional immunogenicity, moving beyond antigen presentation to incorporate receptor-level specificity [68].

Despite these advances, accurately predicting T-cell recognition remains a major unresolved challenge. The TCR-pMHC interaction is governed by highly context-dependent and dynamic structural features that are difficult to generalize across patients. Furthermore, the scarcity of high-quality paired TCR datasets, variability in experimental assays, negative-sample construction, and incomplete representation of population diversity continue to limit model robustness. Benchmarking studies consistently show that current TCR-pMHC or TCR-epitope prediction models remain strongly data-dependent and often struggle to generalize to unseen epitopes [35–37]. As a result, current AI-based approaches are best viewed as tools to refine candidate prioritization rather than definitive predictors of immunogenicity, underscoring the need for continued integration of computational modeling with experimental validation.

### AI-guided vaccine formulation and delivery optimization

For mRNA-based neoantigen vaccines, delivery remains a key translational challenge. AI-guided formulation design is therefore being explored as a way to prioritize lipid nanoparticle (LNP) compositions with desirable delivery properties. These formulations must protect mRNA from degradation, enable efficient cellular uptake and endosomal escape, and balance immune activation with tolerability. These requirements create a multidimensional optimization problem involving lipid chemistry, particle size, surface charge, formulation stability, biodistribution, and transfection efficiency [7, 69, 70].

Machine learning approaches can support this process by prioritizing lipid candidates and formulation parameters before experimental testing. Instead of relying solely on empirical trial-and-error screening, data-driven models can learn relationships between lipid chemical descriptors, formulation composition, and functional readouts such as mRNA encapsulation, cellular uptake, and transfection efficiency. For example, the AGILE platform applies deep learning-guided lipid screening, together with efficient library design and combinatorial chemistry, to identify ionizable lipid candidates associated with improved delivery performance [71]. Similar AI-assisted screening strategies have been used to explore large chemical spaces of ionizable lipids and prioritize candidates for downstream experimental validation, including models designed to predict apparent pKa and mRNA delivery efficiency [72].

More recently, Lipid Optimization using Neural Networks (LiON) applied a directed message-passing neural network to large-scale LNP activity datasets and used the model to screen candidate ionizable lipids in silico. This approach illustrates how deep learning can support the exploration of chemically diverse lipid design spaces before experimental testing [73]. However, AI-guided formulation optimization remains an emerging area rather than a fully mature translational solution. Model performance depends heavily on the quality, scale, and comparability of experimental training datasets, which often differ in cell type, assay conditions, lipid library design, formulation composition, cargo, target tissue, and delivery readouts [74, 75]. Moreover, in vitro transfection efficiency does not necessarily predict in vivo biodistribution, immune activation, or clinical performance [69, 74]. Therefore, AI-based LNP optimization should be viewed as a tool to narrow chemical and formulation search spaces, rather than as a substitute for systematic experimental validation, pharmacokinetic assessment, and safety evaluation.

Taken together, AI-enabled approaches across neoantigen discovery, antigen presentation prediction, TCR-aware prioritization, and formulation optimization can support a more integrated framework for personalized vaccine development. Their near-term value lies in improving the efficiency, reproducibility, and biological rationale of candidate selection across the neoantigen vaccine pipeline.

### Translational synthesis: prediction performance, repertoire design, and prioritization framework

#### Evidence for measurable improvements in neoantigen prediction

AI-enabled methods have improved selected steps of neoantigen prediction, but the magnitude and translational relevance of these improvements depend on the endpoint being evaluated. The most consistent gains have been reported for peptide-HLA binding and natural HLA ligand presentation, whereas functional immunogenicity, TCR recognition, and end-to-end clinical prediction remain more difficult to assess.

For HLA class I binding and presentation, improvements have mainly come from the incorporation of larger binding datasets, mass spectrometry-derived eluted ligand data, antigen-processing-related features, and pan-allelic learning. NetMHCpan-4.0 improved peptide-MHC class I interaction prediction by integrating binding affinity and eluted ligand data, thereby improving the identification of naturally processed ligands, cancer neoantigens, and T-cell epitopes [53]. In a comprehensive benchmark of 18 HLA class I predictors across 32 HLA alleles, MHCflurry achieved an AUC of 0.911 for binding/non-binding classification on synthetic peptides, while NetMHCpan-4.0 also showed strong predictive performance [76]. Importantly, tools trained with eluted ligand data showed better performance for naturally processed MHC ligands than binding-affinity-only predictors [76]. MHCflurry 2.0 further extended class I prediction by incorporating antigen-processing-related features into an integrated presentation model and showed improved performance on held-out mass spectrometry datasets [54]. Together, these studies indicate that the strongest measurable gains have occurred at intermediate presentation-related endpoints, particularly HLA class I binding, antigen-processing-aware presentation prediction, and prioritization of peptides more likely to be naturally presented by HLA molecules.

These improvements in binding and presentation prediction should be distinguished from the more difficult task of predicting functional T-cell immunogenicity and the positive predictive value of selected neoantigens. DeepImmuno is one example of a deep learning-based approach developed to predict immunogenic peptides for T-cell immunity, including applications to viral epitopes and cancer neoantigens [58]. However, independent evaluations using the ITSNdb database of 199 experimentally validated tumor-specific neoantigens showed substantial performance variability among immunogenicity prediction methods, with deep learning predictors ranking highly in some datasets but showing discrepancies across validation datasets [77]. These findings suggest that AI-based tools may improve the ranking of candidate immunogenic neoantigens in benchmark settings, but the extent of improvement in positive predictive value for functional immunogenic neoantigens remains dataset-dependent and requires further validation across diverse patient cohorts and experimental systems.

TCR recognition remains an even more challenging endpoint. TCR-informed tools such as NetTCR-2.0, DeepTCR, TRAP, and SageTCR support receptor-aware or structure-aware prioritization, but current evidence indicates that generalization remains limited. In a systematic benchmark comparison of five TCR-pMHC prediction tools, including TITAN, NetTCR-2.0, ERGO, DLpTCR, and ImRex, model performance was strongly dependent on training data size and balance, and the evaluated models did not generalize well to peptides absent from the training set [36]. Thus, in contrast to HLA presentation prediction, TCR-pMHC prediction has not yet shown the same level of generalizable improvement, particularly for unseen epitopes [37]. Therefore, TCR-informed models are most appropriately used as prioritization or hypothesis-generating tools, with functional validation remaining important.

At the integrated workflow level, tools such as pVACtools, DeepNeo, and NeoDisc illustrate the field’s movement toward combining mutation calling, expression evidence, HLA presentation prediction, antigen-processing features, and immunogenicity-related information into more structured candidate prioritization workflows [9, 49, 59]. These approaches can support more systematic ranking and interpretation, but direct comparison across tools remains difficult because studies differ in training data, benchmark datasets, HLA coverage, immune assays, and whether the evaluated endpoint is binding, natural presentation, T-cell response, or clinical outcome. At present, direct prospective evidence that AI-selected neoantigens improve vaccine-induced immune responses or clinical outcomes over conventional rule-based or expert-curated workflows remains limited.

Taken together, current evidence suggests a clear hierarchy in the maturity of AI-enabled neoantigen prediction. The greatest and most consistent gains have been observed for HLA class I binding and antigen-processing-aware presentation prediction, with benchmark AUC values of approximately 0.91 for binding/non-binding classification and improved performance of eluted ligand-trained models for naturally presented peptides. Immunogenicity-oriented models have begun to improve candidate ranking beyond binding affinity alone, but the extent of improvement in positive predictive value (PPV) for functional immunogenic neoantigens remains dataset-dependent. TCR-pMHC prediction shows limited generalization to unseen epitopes, while integrated workflows can support systematic prioritization but have not yet demonstrated prospective superiority in vaccine-induced immune responses or clinical outcomes. Prospective validation using standardized endpoints for natural presentation, CD4⁺ and CD8⁺ T-cell responses, response breadth, durability, and clinical outcomes will be required to determine whether AI-guided selection improves end-to-end vaccine performance.

#### An effective neoantigen repertoire: breadth, quality and feasibility

An effective personalized neoantigen vaccine repertoire should not be defined by the number of candidate neoantigens alone. It should instead be considered in relation to tumor relevance, HLA coverage, CD4⁺ and CD8⁺ response potential, vaccine platform, and manufacturing feasibility. As summarized in Table 1, published studies have used variable repertoire sizes across peptide, RNA, DNA, viral-vector, and dendritic-cell-based platforms. These designs range from fewer than 10 candidates in some studies to 30 or more candidates in others, with some viral-vector platforms [12, 15, 16, 19, 27] accommodating substantially larger repertoires (Table 1). These numbers should be interpreted as platform- and study-specific design choices; current clinical data do not establish a universal optimal threshold (Table 1) [1, 2]. Including more candidates may increase the chance of covering multiple tumor clones, HLA alleles, and CD4⁺/CD8⁺ response pathways. However, current evidence does not show a consistent relationship between epitope number alone and improved immunogenicity or clinical benefit. Not all predicted or selected neoantigens are naturally presented or immunogenic, and response rates vary across epitopes, patients, assays, and vaccine platforms. Larger repertoires may also introduce lower-confidence candidates, increase construct complexity, complicate quality control, prolong manufacturing, dilute antigen dose within a fixed delivery format, or increase the burden of immune monitoring. Thus, repertoire breadth should reflect biologically meaningful coverage rather than numerical abundance.

At present, no clinically validated minimal or optimal number of neoantigens has been established (Table 1) [1, 2]. A smaller repertoire enriched for clonal, highly expressed, naturally presented, and immunogenic candidates may be more informative than a larger repertoire composed mainly of candidates supported only by prediction scores. Conversely, tumors with higher mutation burden or greater intratumoral heterogeneity may require broader antigen coverage to reduce immune escape. Treatment setting may also matter, because patients treated in the adjuvant or minimal residual disease setting may differ from those with advanced or rapidly progressing disease in immune competence, disease kinetics, and the urgency of vaccine manufacturing. Therefore, repertoire size should be determined by candidate quality and diversity, tumor type and mutation burden, clonal architecture, HLA class I and II coverage, CD4⁺ and CD8⁺ response potential, normal-tissue exclusion, platform capacity, and clinically actionable turnaround time.

The most informative endpoints are not only the number of neoantigens selected, but also the proportion of selected, manufactured, administered, or immune-evaluable candidates that induce measurable T-cell responses, and the proportion of patients who develop broad or multi-epitope vaccine-specific responses. Epitope spreading to non-vaccine antigens, when reported, may provide additional evidence of broader antitumor immune activation, but should be distinguished from direct vaccine-specific multi-epitope responses. Because these denominators are not consistently reported across studies, direct cross-trial comparison remains limited. We therefore summarize available data on candidate number, manufacturing feasibility, turnaround time, immune-evaluable antigens, and reported response breadth in Table 1.

#### Toward an evidence-weighted portfolio and closed-loop prioritization framework

To bridge the gap between individual computational tools and translational implementation, a practical strategy for AI-enabled personalized neoantigen vaccine development is unlikely to depend on a single algorithm or software package. Given the biological uncertainty introduced at each step of the pipeline, a more practical strategy is an evidence-weighted, platform-aware, and closed-loop prioritization framework. In this framework, neoantigen selection is approached as the construction of a biologically supported, manufacturable, and experimentally verifiable antigen portfolio rather than the ranking of individual peptides alone [3, 78]. To make this strategy more actionable, we propose a multi-stage decision-gate framework for assembling personalized neoantigen repertoires (Box 1).

The individual components of this framework are grounded in existing biological, computational, and clinical evidence. Candidate generation should begin with canonical somatic SNVs, indels, frameshift variants, and fusions, while selected noncanonical antigen sources may be incorporated when supported by transcriptomic, proteogenomic, or immunopeptidomic evidence [51, 52, 79]. Tumor relevance filtering is supported by recommendations to consider variant allele fraction, expression, clonality, and tumor specificity in neoantigen prioritization [3]. Presentation modeling should integrate HLA binding, antigen-processing-aware features, pan-allelic prediction, and eluted-ligand or immunopeptidomic evidence, while recognizing persistent limitations for HLA class II prediction and rare or underrepresented HLA alleles [61, 78, 80]. Immunogenicity refinement may incorporate peptide foreignness, self-similarity, peptide abundance, pMHC structural features, and TCR-informed information, but these features should be used to refine candidate ranking rather than replace functional validation [81]. Portfolio-level selection should then balance candidate quality, clonal and HLA coverage, CD4⁺/CD8⁺ response potential, platform capacity, manufacturability, and clinically actionable turnaround time, as discussed above and summarized in Table 1.

This framework should be viewed as a translational decision-support strategy rather than a validated clinical solution. Although each decision gate is grounded in current biological and computational evidence, the proposed six-gate structure itself has not been prospectively validated or directly compared with alternative organizational frameworks. Its future implementation will require standardized assay definitions, harmonized immune-response endpoints, traceable software versions, linked manufacturing records, and prospective benchmarking across clinically relevant endpoints, including not only immunogenicity but also durable clinical outcomes.

Box 1. Proposed decision-gate framework for AI-enabled neoantigen prioritization Gate 1: candidate generation

Generate canonical candidates from tumor-specific somatic SNVs, small insertions or deletions, frameshift variants, and gene fusions when applicable. In parallel, incorporate noncanonical antigen sources, such as aberrant splicing, intron retention, alternative ORFs, circRNA-derived products, and transposable-element-derived antigens, when supported by transcriptomic, proteogenomic, or immunopeptidomic evidence.

Gate 2: tumor relevance and specificity filtering

Prioritize candidates with stronger tumor relevance by integrating tumor expression, variant clonality, allele-specific expression when available, tumor specificity, and normal-tissue exclusion. When longitudinal or multi-region data are available, this step should also consider intratumoral heterogeneity and potential immune-escape mechanisms, such as antigen loss or impaired HLA presentation.

Gate 3: HLA presentation evidence

Evaluate HLA class I and class II presentation using binding prediction, antigen-processing-aware features, pan-allelic modeling, and eluted-ligand or immunopeptidomic evidence when available. Prediction results should be interpreted cautiously for HLA class II molecules and rare or underrepresented HLA alleles, where training data and model coverage remain limited.

Gate 4: immunogenicity refinement

Refine candidates using features related to peptide foreignness, self-similarity, peptide abundance, pMHC structural features, and TCR-informed prediction when sufficiently supported by available data. These features should be used to refine prioritization rather than to replace functional immune validation.

Gate 5: portfolio-level selection

Select the final antigen set as a coordinated repertoire rather than a list of isolated high-scoring peptides. The aim is to balance clonal tumor coverage, HLA-I and HLA-II diversity, CD4⁺ and CD8⁺ response potential, low redundancy, platform compatibility, and manufacturability.

Gate 6: manufacturability and clinical delivery

Evaluate the selected antigen portfolio against platform-specific constraints, including sequence compatibility, synthesis feasibility, construct design, delivery format, payload or design limits, quality-control requirements, and clinically actionable turnaround time.

Feedback loop: experimental and clinical learning

Link post-vaccination immune-monitoring data, such as ELISpot, intracellular cytokine staining, multimer assays, TCR sequencing, single-cell profiling, immunopeptidomics when available, manufacturing records, and clinical outcomes, back to the original computational features. These data can support future model refinement and help identify which candidate features are associated with presentation, T-cell recognition, response breadth, durability, and clinical benefit.

#### Future perspectives and implementation considerations

The future of neoantigen cancer vaccine development will likely depend on the closer integration of computational modeling, immunogenomics, formulation science, and prospective clinical validation. As discussed above, current AI-enabled approaches can support candidate prioritization across multiple stages of the vaccine pipeline, but their translational value will ultimately depend on whether model predictions are reproducible, biologically interpretable, and prospectively validated in clinically relevant settings.

#### Prospective benchmarking and clinical validation

Future studies should move beyond retrospective benchmarking and incorporate standardized evaluation frameworks. It will be important to assess not only peptide-HLA binding or presentation prediction, but also downstream endpoints such as natural antigen presentation, T-cell recognition, vaccine-induced immune responses, and clinical outcomes [35]. Integrating computational innovation with rigorous immunological and clinical validation will be essential for determining whether AI-enabled prioritization can meaningfully improve the clinical performance of personalized neoantigen vaccines.

#### Data diversity and explainable AI

Future neoantigen prediction models will require more diverse and representative training datasets. Current models remain limited by uneven coverage of immunopeptidomics data, paired TCR-pMHC datasets, and HLA-specific training examples across populations and tumor types. Although pan-allelic methods may partially mitigate these gaps, the limited availability of binding and immunological data for some HLA alleles remains a concern for equitable model development and evaluation [41, 42]. Larger multi-institutional datasets, harmonized immunological assays, and privacy-preserving learning frameworks may help improve model generalizability while protecting patient-level data. In parallel, uncertainty-aware and explainable AI approaches will be important for clinical adoption. Such frameworks can guide investigators in understanding why specific neoantigens are prioritized and how to interpret model confidence in the context of experimental validation [82].

#### Regulatory traceability and adaptive implementation

With the accelerating advancement of artificial intelligence in neoantigen cancer vaccine development, regulatory science must evolve accordingly to address the challenges of these highly personalized therapies. Recently, the UK Medicines and Healthcare products Regulatory Agency (MHRA) released draft guidance for individualized mRNA cancer immunotherapies [83]. The term “immunotherapies” is deliberately used instead of “vaccines” because regulatory authorities classify infectious disease vaccines and therapeutic cancer vaccines under different regulatory frameworks. The UK MHRA proposes life-cycle regulatory guidance, from the design phase through post-marketing monitoring and public communication. Specifically, AI and machine learning algorithms used for neoantigen selection may qualify as Software as a Medical Device, depending on the exact techniques, processes, and applicable jurisdictional interpretations [83]. This is particularly relevant when software analyzes genetic data, predicts treatment suitability, or creates individualized treatment designs based on patient biodata. Manufacturers are expected to adhere to Good Machine Learning Practice when developing and using these algorithms. Developers may also be required to provide transparent descriptions of the analytical pipeline, algorithm performance metrics, and evidence that training datasets are representative of the intended target patient populations.

Ensuring traceability will be one of the most fundamental requirements for product validation. Manufacturers will need robust mechanisms, such as unique patient identifiers and barcode systems, to link specific versions of computational pipelines and software to the patient’s original genomic data, the selected neoantigen sequences, the manufactured product, and the administered dose. For marketing authorization, manufacturers may need to include AI and machine learning version tracking within their risk management plans. Any software updates will require rigorous documentation and assessment of how computational changes may affect clinical safety or efficacy. Therefore, the future of personalized neoantigen vaccines will rely not only on scientific and technological innovation, but also on how regulatory science evolves to accommodate these novel, computationally guided therapies.

## Conclusions

Personalized neoantigen cancer vaccines offer a rational strategy for directing immune responses against tumor-specific, patient-derived antigens. However, their clinical value depends on more than the identification of tumor mutations. Candidate antigens must be expressed, processed, presented by HLA molecules, recognized by T cells, incorporated into an appropriate vaccine formulation, and delivered within a clinically meaningful timeframe. Losses at any of these steps can reduce the likelihood that a selected candidate will translate into a functional immune response.

AI-enabled approaches will  improve this process by making candidate prioritization more systematic and biologically informed. Multi-omics integration can strengthen the evidence supporting antigen selection, antigen-processing and HLA-presentation models can refine candidate filtering, structure- and TCR-aware methods may help prioritize epitopes with greater immunological relevance, and machine learning-based formulation strategies can support the exploration of lipid and delivery design spaces. These approaches are most useful when they are embedded within an experimentally grounded workflow, where computational predictions are linked to immunopeptidomics, functional T-cell assays, manufacturing quality control, and clinical immune monitoring.

The next stage of cancer vaccine discovery requires prospective benchmarking, more representative training datasets, interpretable model outputs, and traceable computational and manufacturing processes. Rather than serving as a stand-alone solution, AI is likely to be most valuable as part of an integrated translational framework that connects antigen discovery, biological validation, formulation development, and regulatory oversight. With such integration, personalized neoantigen vaccines may become more feasible and reproducible components of precision oncology.

## Data Availability

No datasets were generated or analysed during the current study.

## Abbreviations

AI: Artificial intelligence
ANN: Artificial neural network
CDR3: Complementarity-determining region 3
DNA: Deoxyribonucleic acid
GNN: Graph neural network
HCC: Hepatocellular carcinoma
HLA: Human leukocyte antigen
HNSCC: Head and neck squamous cell carcinoma
HPV: Human papillomavirus
HR: Hazard ratio
LNP: Lipid nanoparticle
LSTM: Long short-term memory
MHRA: Medicines and Healthcare products Regulatory Agency
MHC: Major histocompatibility complex
ML: Machine learning
mRNA: Messenger ribonucleic acid
MS: Mass spectrometry
NSCLC: Non-small cell lung cancer
NSTC: National Science and Technology Council
ORR: Objective response rate
PD-1: Programmed cell death protein 1
PDAC: Pancreatic ductal adenocarcinoma
PLM: Protein language model
pMHC: Peptide-major histocompatibility complex
RFS: Recurrence-free survival
RNA: Ribonucleic acid
RNA-seq: RNA sequencing
TAP: Transporter associated with antigen processing
TCR: T-cell receptor
V/D/J: Variable, diversity, and joining (genes)
WES: Whole-exome sequencing
